# A Study to Evaluate the Effect of Sodium-Glucose Co-transporter 2 (SGLT2) Inhibitors on Oxidative Stress Parameters in Type 2 Diabetes Mellitus Patients

**DOI:** 10.7759/cureus.58536

**Published:** 2024-04-18

**Authors:** Ankita Sharma, D Aruna, Anne Beatrice

**Affiliations:** 1 Department of Clinical Pharmacology and Therapeutics, Nizam's Institute of Medical Sciences, Hyderabad, IND; 2 Department of Endocrinology, Nizam's Institute of Medical Sciences, Hyderabad, IND

**Keywords:** lipid profile, glycemic parameters, sglt-2 inhibitors, oxidative stress, diabetes mellitus

## Abstract

Introduction

Diabetes mellitus (DM) is a global health issue with 50 million diabetics currently residing in India. Hyperglycemia causes tissue damage due to mitochondrial overproduction of reactive oxygen species. Sodium-glucose cotransporter-2 (SGLT2) inhibitors (SGLT2i) have shown a decrease in oxidative stress by either amelioration of free‐radical generation or potentiation of cellular antioxidative capacity in preclinical studies. However, there is a paucity of published clinical studies. Hence, this study was undertaken to evaluate the effect of co-administration of SGLT2i with other drugs on oxidative stress in type 2 DM (T2DM) patients.

Methods

A prospective, parallel, open-label study in T2DM patients attending endocrinology OPD was conducted for a period of 12 months. At the clinician’s discretion, patients were grouped as SGLT2i as an add-on to standard drugs vs standard drugs alone. Blood samples were collected at baseline and at the end of 12 weeks to estimate malondialdehyde (MDA), nitric oxide (NO), and glutathione (GSH) levels. Secondary parameters - glycemic indices and lipid profile - were estimated every four weeks.

Results

A total of 32 patients were enrolled in the study (16 per group). There was a significant decrease in MDA (p < 0.05) and NO (p < 0.01) and a highly significant increase in GSH (p < 0.001) at 12 weeks from baseline in the SGLT2i group. A reduction in fasting blood sugar (FBS) and post-prandial blood sugar (PPBS) and a 0.56% difference in HbA1c were also noted in the SGLT2i group. Significant lowering of low-density lipoprotein (LDL, p < 0.05) and elevation in HDL levels (p < 0.05) from baseline was seen in the SGLT2i group.

Conclusion

Co-administration of SGLT2i with antidiabetic drugs demonstrated a significant effect in improving oxidative stress biomarkers and glycemic and lipid profiles among T2DM patients.

## Introduction

Diabetes is a complex, chronic illness due to the lack of insulin secretion (type 1 diabetes mellitus, T1DM) or a decrease in insulin sensitivity of tissues to insulin (type 2 diabetes mellitus, T2DM) with impaired carbohydrate, fat, and protein metabolism. T2DM is a growing epidemic of all ages in developing countries [1]. According to various population-based surveys, the prevalence of T2DM in India was reported to be between 10.2% and 36% [2]. The World Health Organization (WHO) estimated that the global prevalence of diabetics is expected to reach 300 million by 2025 and 366 million by 2030 [3]. According to the International Diabetes Federation (IDF), the number of diabetic cases will rise to 642 million by 2040 [4]. The burden of disease is increasing worldwide due to sedentary lifestyles, obesity, and unhealthy dietary habits.

Oxidative stress is involved in the development and progression of diabetes complications and may result from either increased free‐radical production, a reduction in antioxidative capacity, or a combination of both. T2DM is associated with multiple microvascular and macrovascular complications. Oral antidiabetic drugs with antioxidative potential are beneficial for these patients. The studied oral antidiabetic drugs for antioxidative effects include metformin, thiazolidinediones [5], and gliclazide [6]. Metformin, which is the first-line antidiabetic drug, showed some antioxidative potential in vitro studies, but the results with in vivo and human studies showed no effect on oxidative stress parameters [7-10].

The novel oral hypoglycaemic agents sodium-glucose co-transporter 2 (SGLT2) inhibitors (SGLT2i) (canagliflozin, empagliflozin, and dapagliflozin) act by inhibiting SGLT2 receptor protein on proximal tubules of kidneys. The role of SGLT2 inhibition in preventing or reducing oxidative stress states has been explored in several animal studies over the past decade. In a study by Abdel-Wahab et al. [8], kidneys of diabetic rats treated with dapagliflozin showed lower levels of the lipid oxidation product, malondialdehyde (MDA), and higher activities of antioxidant enzymes, glutathione peroxidase (GH-Px) and superoxide dismutase (SOD). The study by Cheng et al. in streptozotocin-induced type 1 diabetic rats has shown a reduction in reactive oxygen species (ROS) in pancreatic β-cells by the use of empagliflozin [9]. Another study done in streptozotocin-induced T1DM rats revealed that empagliflozin can prevent endothelial dysfunction in aortic rings and alleviate oxidative stress in aortic vessels [10]. A study by Tahara et al. on a high-fat diet and streptozotocin-nicotinamide-induced type 2 diabetic mice exhibited increases in the levels of oxidative stress, inflammation markers, and aminotransferase, which were dose-dependently and significantly decreased by ipragliflozin, suggesting that ipragliflozin can lower diabetes-induced oxidative stress and inflammation, thus attenuating hepatic injury [11]. Osorio et al. also concluded that SGLT2 inhibition by phlorizin use prevents oxidative stress in the kidneys of diabetic rats, suggesting a pro-oxidative mechanism linked to SGLT2 action [12]. Dapagliflozin has shown an important antioxidant-like cardio-protective effect in Met S-rats, in some aspects similar to the insulin effect, affecting Zn2+-regulation via Zn2+-transporters, MMPs, and oxidative-stress connective tissue growth factor in the diabetic kidney [13].

A study by Iannantuoni et al. provided consistent and novel evidence of the antioxidant effect of empagliflozin treatment in humans [14]. Another human study showed that dapagliflozin promoted a reduction in urinary -OHdG: 8-hydroxy-2′-deoxyguanosine excretion, an indirect marker of oxidative stress [15,16]. Multiple studies over the last decade have shown that SGLT2 inhibition has the potential to counteract this oxidative stress. However, most studies are in animal models with very few published data regarding the antioxidative effect of SGLT2i in humans. Other studies have not evaluated serum biomarkers of oxidative stress in diabetic patients. Hence, the present study was planned to evaluate the antioxidative properties of SGLT2i by assessing the oxidative stress parameters in T2DM patients.

## Materials and methods

The study was conducted at the Department of Clinical Pharmacology and Therapeutics in collaboration with the Department of Endocrinology, Nizam’s Institute of Medical Sciences, Hyderabad, Telangana State, India. The study protocol was approved by the NIMS Institutional Ethics Committee (Approval number EC/NIMS/2562/2020) and registered at the Clinical Trial Registry India (CTRI No. CTRI/2020/11/028806). Written informed consent was taken from all the subjects. The study was a prospective, open-label, parallel, single-center comparative pilot study conducted from November 2020 to November 2021. The treatment duration was three months. However, the sample size of 40 patients (20 patients in each group) could not be completed due to the COVID-19 pandemic, and we only recruited 32 patients. The inclusion criteria were T2DM patients aged between 30 and 60 years of either gender with a duration of disease ≥ three years, HbA1c between 7.0 and 9.5 %, and who were SGLT2i-naïve patients on a stable dose of two or three oral antidiabetic drug combinations for the past three months. The allowed antidiabetic combinations were metformin ± sulphonyl urea ± gliptins. Exclusion criteria were a history of hypersensitivity to the study drugs; patients using glucagon-like peptide-1 agonists or insulin in the past 12 weeks; patients with T1DM; patients with history of liver disease (alanine aminotransferase, alkaline aminotransferase, or alkaline phosphatase levels > three times the upper limit of normal) or kidney disease (serum creatinine > 1.1 mg/dL) or a history of chronic smoking or alcohol addiction; and pregnant and lactating women.

Eligible subjects were enrolled in the study. After baseline investigations, all the patients were divided into two groups. Group I (treatment group) received standard antidiabetic medications along with SGLT2i, while Group II (control group) received only standard antidiabetic medications as per clinician’s discretion. The SGLT2i allowed in the study were canagliflozin (100-300 mg/day) or dapagliflozin (5-10 mg/day) or empagliflozin (10-25 mg/day) or remogliflozin (100 mg/day to be taken once daily with breakfast). The treatment period was 12 weeks. The primary endpoint was to assess any change in oxidative stress markers, which included MDA, nitric oxide (NO), and glutathione levels at the end of 12 weeks. The secondary endpoints were to assess any change in HbA1c, fasting blood sugar (FBS), and post-prandial blood sugar (PPBS), total cholesterol, triglycerides, high-density lipoprotein concentration (HDL), and low-density lipoprotein concentration (LDL) from baseline and to study the incidence of adverse effects in the study population. Follow-up visits were planned at zero, four, eight, and 12 weeks of treatment. During each visit weight, systolic blood pressure (SBP), diastolic blood pressure (DBP), FBS, and PPBS were measured. Oxidative parameters - MDA, NO and glutathione, HbA1c, total cholesterol, triglycerides, HDL, and LDL, were measured at zero and 12 weeks. Compliance was measured by the pill count method. Patients requiring additional treatment or dose adjustments outside the pre-specified ranges; patients with persisting abnormality, which, in the opinion of the investigator/clinician, makes the participation risky; or patients who were not compliant (not taking at least 80% of the prescribed medication) were withdrawn from the study. Safety assessments were done at every visit.

Statistical analysis

Data were presented as mean ± standard deviation. Some of the study variables were non-normally distributed, as determined by the Shapiro-Wilk test, and presented as median with an inter-quartile range. Comparison within the group was performed by student t-test and repeated measures ANOVA for normally distributed data and Wilcoxon test and Friedman test for non-normal data, followed by post-hoc analysis by Holm-Sidak’s test. Comparison between groups was performed by the unpaired t-test for normally distributed data and the Mann-Whitney U test for non-normal data. Missing data were handled by the last observation carried forward (LOCF). All the subjects who completed at least one efficacy assessment were considered for efficacy analysis. All subjects who were enrolled in the study were considered for safety analysis. Safety data were expressed as proportions. P ≤ 0.05 was considered as statistically significant. Statistical analysis was performed using the Graph Pad Prism (version 5; 2020 Graph Pad Software, Inc., La Jolla, CA).

## Results

**Figure 1:**
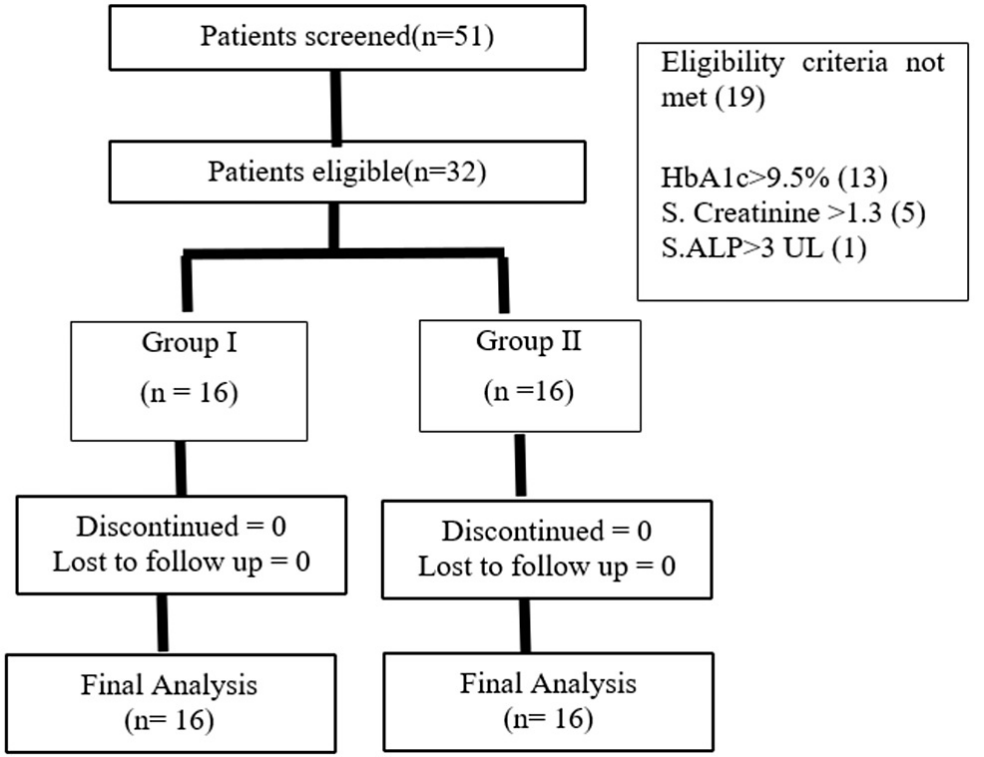
Participant flow diagram Group I: Standard antidiabetic medications + SGLT2 inhibitor; Group II: Standard antidiabetic medications

A total of 51 patients were screened. Out of 51 patients, 19 patients were screen failures (Figure 1). In the final analysis 32 patients with 21 males and 11 females were in cluded. The mean age of the participants was 50.28 ± 10.47 years. The other demographics characteristics are given in Table 1.

**Table 1:** Demographic characteristics of the study groups Group I: Standard antidiabetic medications+ SGLT2 inhibitor; Group II: Standard antidiabetic medications Data presented as mean ± standard deviation Comparison between groups performed by the unpaired t-test * P < 0.05 compared to Group II

Characteristic	Group I (n=16)	Group II (n=16)
Sex (M:F)	10:6	11:5
Age (yrs.)	50.81 ± 10.21	49.75 ± 11.05
Weight (kg)	70.25 ± 10.88*	72 ± 9.26
Duration of diabetes (yrs)	6.75 ± 2.62	6.62 ± 3.18
SBP (mmHg)	128.1 ± 10.86	133.4 ± 11.42
DBP (mmHg)	85.8 ± 8.87	87.5 ± 7.06

Primary outcome analysis

There was a significant decrease (p < 0.05) in MDA at 12 weeks (27.7%) from baseline in Group I, while an increase in MDA (12.8%) was observed in Group II (Table 2). There was no significant decrease in MDA between the groups at 12 weeks.

**Table 2:** Primary outcome analysis Group I: Standard antidiabetic medications+ SGLT2 inhibitor; Group II: Standard antidiabetic medications Data presented as median with the inter-quartile range Comparison within the group performed by the Wilcoxin test and Friedman test; comparison between groups performed by Mann Whitney U test. * P < 0.05 compared to Group II; * P < 0.05 compared to baseline; ** P < 0.01 compared to baseline; *** P < 0.001 compared to baseline

Parameter	Group I, 0 week	Group I, 12 weeks	P value	Group II, 0-week	Group II, 12 weeks	P value
MDA (nM/mL)	13.71 (10.00-27.33)	9.9 (8.54-17.63)	0.0476*	11.68 (10.28-18.97)	13.18 (11.87-16.99)	0.7869
NO (µM/L)	13.83 (10.11-17.75)	11.00 (10.02-12.32)	0.0078**	12.33 (9.99-26.8)	12.5 (9.49-26.82)	0.583
GSH (µM/L)	543.3 (518.3-646.0)	643.5 (615.0-707.1)	0.0003***	636.3 (560.5-695.4)	636.2 (542.5-682.1)	0.3652

A significant decrease (p < 0.01) was found in NO levels at 12 weeks (20.46%) from baseline in Group I, while a little increase (1.37%) was observed in Group II (Table 2). There was no significant difference in NO levels between the groups at the end of 12 weeks.

There was a highly significant increase (p < 0.001) in glutathione levels at 12 weeks from baseline in Group I (18.44%) and no significant increase in Group II (Table 2). Moreover, there was a 0.015% decrease from baseline in Group 2. No statistically significant difference was observed in glutathione levels between the groups.

Secondary outcome analysis

There was a statistically significant (p < 0.01) decrease in FBS at the end of 12 weeks in Group I compared to baseline, whereas in Group II, no significant decrease was noted (Table 3). No significant change was found between the groups. A statistically significant (p < 0.01) decrease in PPBS was observed in Group I compared to baseline, while no significant decrease was noted in Group II at the end of 12 weeks. There was also a significant (p < 0.01) decrease in PPBS in between the groups at 12 weeks (Table 3). A statistically significant decrease in HbA1c was found in both the study groups, Group I (p < 0.0001) and Group II (p < 0.05) at the end of 12 weeks compared to baseline (Table 3). In between group comparison showed no significant difference in HbA1c at the end of 12 weeks.

**Table 3:** Secondary outcome analysis Group I: Standard antidiabetic medications + SGLT2 inhibitor; Group II: Standard antidiabetic medications Data presented as mean ± SD Comparison within the group performed by Student's t-test and repeated measures ANOVA Comparison between groups performed by the unpaired t-test * P < 0.05 compared to baseline; ** P < 0.01 compared to baseline; *** P < 0.0001 compared to baseline; $ P < 0.01 compared to Group II

Parameter	Group I, 0 week	Group I, 12 weeks	P value	Group II, 0 week	Group II, 12 weeks	P value
FBS (mg/dL)	147.6 ± 35.1	123.7 ± 24.2	0.0081**	142.7 ± 43.8	125 ± 30.6	0.1156
PPBS (mg/dL)	207.9 ± 48.8	172.3 ± 50.3	0.0011** $	200 ± 63.7	181.9 ± 48.5	0.3523
HbA1c (%)	7.86 ± 0.78	7.31 ± 0.75	< 0.0001***	7.9 ± 0.7	7.5 ± 0.8	0.018*
T Cholesterol (mg/dL)	149.4 ± 26.2	143.5 ± 22.9	0.1758	159.8 ± 34	153.7 ± 33.4	0.4380
LDL (mg/dL)	85.00 ± 13	78 ± 18.23	0.0408*	89.13 ± 28.6	88.38 ± 27.5	0.8836
HDL (mg/dL)	37.19 ± 6.4	39.81 ± 6.8	0.0396*	40.1 ± 1.73	40.2 ± 5	0.9060
Triglycerides (mg/dL)	133.3 ± 35.1	127.5 ± 29.6	0.1075	154.3 ± 45.3	168.3 ± 44.3	0.3094
Weight (kg)	70.25 ± 10.8	68.38 ± 11.0	<0.0001***	72.00 ± 9.26	72.50 ± 9.95	0.4754

We observed no significant change in the total cholesterol levels in both the study groups at the end of 12 weeks compared to baseline (Table 3). In-between group comparison also showed no significant change in total cholesterol at the end of 12 weeks. There was a statistically significant (p < 0.05) decrease in LDL and an increase in HDL levels in Group I at the end of 12 weeks as compared to baseline (Table 3). No significant change was found in Group II in these parameters. In between the study groups, no significant difference was observed in LDL and HDL levels. There was no statistically significant change in triglyceride levels in the groups at the end of 12 weeks as compared to that of baseline. However, in-between group comparison showed a statistically significant change (p < 0.01) in triglyceride levels at the end of 12 weeks. We observed a statistically significant decrease in weight in Group I at the end of 12 weeks (p < 0.0001) as compared to that of Group II.

Safety analysis

A total of 22 adverse events were noted in 32 patients. There were no serious adverse drug reactions during the study. The most common adverse effects in Group I (SGLT2i) were fatigue/tiredness, giddiness, leg cramps, sweating, weight loss, increased urination, and thirst. These symptoms were mild and self-limiting. All the adverse reactions were managed symptomatically without stopping treatment, with adequate rest and food. There was no statistically significant difference in the incidence of adverse reactions between the groups. The study drugs were tolerated well.

## Discussion

In this present study, we tried to explore the antioxidative potential of SGLT2i using three oxidative biomarkers, namely, MDA, NO, and glutathione, when co-administered with other oral antidiabetics in T2DM patients. We found a significant decrease in prooxidants, MDA, and NO and a significant elevation in GSH levels, which are antioxidants, after the addition of SGLT2i to standard antidiabetic drugs. These results were similar to the study conducted by Iannantuoni et al., which demonstrated that T2DM patients receiving SGLT2i treatment undergo a reduction in mitochondrial superoxide production in parallel to an increase in glutathione content providing evidence of the antioxidant effect of empagliflozin treatment in humans [14]. Another study by Shigiyama et al. named DEFENCE trial showed that the dapagliflozin add-on to metformin had improvement in oxidative stress by a significant reduction in the levels of urinary oxidative biomarker 8-hydroxy-2′-deoxyguanosin [16]. A study by Nabrdalik-Leśniak et al. demonstrated that SGLT2i treatment affects the urine antioxidant status by improvement of the SOD antioxidant activity in T2DM patients, which could be the mechanism behind its nephroprotective activity [17]. Another study by Lambadiari et al. demonstrated that 12-month treatment with GLP-1RA, SGLT2i, and their combination provided a significant reduction of MDA but an improvement in serum levels of antioxidant biomarkers, which was more prominent and appeared earlier (at four months) in the GLP1-RA+SGLT2i group [18].

We observed a significant change in HbA1c, FBS, and PPBS in the SGLT2i group. Our findings were consistent with the results of previous studies. Bashier et al.'s study in T2DM patients after administration of dapagliflozin (10 mg) or canagliflozin (100 g) for six months found a 0.9% decrease in HbA1c as compared to our study where we found a 0.56% difference in HbA1c at the end of three months [19]. Strojek et al.’s study in uncontrolled type 2 diabetics on glimepiride background therapy showed a significant reduction (0.82%) in HbA1c after 24 weeks of dapagliflozin therapy compared to that of placebo (0.13%) [20]. We found a significant reduction in FBS and PPBS at 12 weeks from baseline in the SGLT2i group as compared to that of the control group. A retrospective study by Panikar et al. on the efficacy of SGLT2i in the management of uncontrolled T2DM showed a greater reduction in hyperglycemia [21].

In our study, we found a significant reduction in LDL and elevation in HDL levels at 12 weeks compared to baseline in the SGLT2i group. The SGLT2i use improved two treatment goals, which are the targets for the management of dyslipidemia in diabetic patients. Previous studies on SGLT2i have reported inconsistent results regarding their effect on lipid profile. A retrospective study assessing the effect of SGLT2i on the lipid profile in T2DM patients observed a significant reduction in total cholesterol (17.6 mg/dL), LDL cholesterol (13.4 mg/dL), and triglyceride levels (25.9 mg/dL) [22]. Our results were consistent with the above study. A significant decrease in weight (1.8 kg) at the end of the study from baseline was noted in the SGLT2i study group. This observation was similar to a previous study by Bashier et al., which showed a statistically significant change of 1.5 kg in weight at the end of six months from baseline [22]. The strength of the study was that all the eligible subjects enrolled in the study could be followed up till the end of the study period. There were a few limitations to the study. We were unable to complete the sample size due to the COVID-19 pandemic, and, hence, the sample size was small.

## Conclusions

Diabetes is a chronic illness and oxidative stress is involved in the development and progression of diabetes complications. Antidiabetic drugs with antioxidative potential will help in dealing with multiple micro and macrovascular complications. Co-administration of SGLT2i along with antidiabetic drugs showed a significant improvement in oxidative stress biomarkers and glycemic and lipid profiles among T2DM patients. Therefore, the addition of SGLT2i could possibly prevent the development of various complications by decreasing the oxidative stress in the body.

Multiple studies in animal models over the last decade have shown that SGLT2 inhibition has the potential to counteract oxidative stress. However, very few published data are available regarding the antioxidative effect of SGLT2i in humans. Further studies with larger sample sizes and longer study duration are required to confirm our findings.

## References

[1] American Diabetes Association (2020). 6. Glycemic targets: standards of medical care in diabetes-2020. Diabetes Care.

[2] Kulkarni S, Kondalkar S, Mactaggart I (2019). Estimating the magnitude of diabetes mellitus and diabetic retinopathy in an older age urban population in Pune, western India. BMJ Open Ophthalmol.

[3] Einarson TR, Acs A, Ludwig C, Panton UH (2018). Prevalence of cardiovascular disease in type 2 diabetes: a systematic literature review of scientific evidence from across the world in 2007-2017. Cardiovasc Diabetol.

[4] Roglic G (2016). WHO Global report on diabetes: a summary. IJNCD.

[5] Bagi Z, Koller A, Kaley G (2004). PPARgamma activation, by reducing oxidative stress, increases NO bioavailability in coronary arterioles of mice with type 2 diabetes. Am J Physiol Heart Circ Physiol.

[6] O’Brien RC, Luo M, Balazs N (2000). In vitro and in vivo antioxidant properties of gliclazide. J Diab Compl.

[7] Ruggiero-Lopez D, Lecomte M, Moinet G, Patereau G, Lagarde M, Wiernsperger N (1999). Reaction of metformin with dicarbonyl compounds. Possible implication in the inhibition of advanced glycation end product formation. Biochem Pharmacol.

[8] Abdel-Wahab AF, Bamagous GA, Al-Harizy RM, ElSawy NA, Shahzad N, Ibrahim IA, Ghamdi SS (2018). Renal protective effect of SGLT2 inhibitor dapagliflozin alone and in combination with irbesartan in a rat model of diabetic nephropathy. Biomed Pharmacother.

[9] Cheng ST, Chen L, Li SY, Mayoux E, Leung PS (2016). The effects of empagliflozin, an SGLT2 inhibitor, on pancreatic β-cell mass and glucose homeostasis in type 1 diabetes. PLoS One.

[10] Aroor AR, Das NA, Carpenter AJ (2018). Glycemic control by the SGLT2 inhibitor empagliflozin decreases aortic stiffness, renal resistivity index and kidney injury. Cardiovasc Diabetol.

[11] Tahara A, Kurosaki E, Yokono M (2013). Effects of SGLT2 selective inhibitor ipragliflozin on hyperglycemia, hyperlipidemia, hepatic steatosis, oxidative stress, inflammation, and obesity in type 2 diabetic mice. Eur J Pharmacol.

[12] Osorio H, Coronel I, Arellano A (2012). Sodium-glucose cotransporter inhibition prevents oxidative stress in the kidney of diabetic rats. Oxid Med Cell Longev.

[13] Olgar Y, Turan B (2019). A sodium-glucose cotransporter 2 (SGLT2) inhibitor dapagliflozin comparison with insulin shows important effects on Zn(2+)-transporters in cardiomyocytes from insulin-resistant metabolic syndrome rats through inhibition of oxidative stress (1). Can J Physiol Pharmacol.

[14] Iannantuoni F, M de Marañon A, Diaz-Morales N (2019). The SGLT2 inhibitor empagliflozin ameliorates the inflammatory profile in type 2 diabetic patients and promotes an antioxidant response in leukocytes. J Clin Med.

[15] Solini A, Giannini L, Seghieri M, Vitolo E, Taddei S, Ghiadoni L, Bruno RM (2017). Dapagliflozin acutely improves endothelial dysfunction, reduces aortic stiffness and renal resistive index in type 2 diabetic patients: a pilot study. Cardiovasc Diabetol.

[16] Shigiyama F, Kumashiro N, Miyagi M, Ikehara K, Kanda E, Uchino H, Hirose T (2017). Effectiveness of dapagliflozin on vascular endothelial function and glycemic control in patients with early-stage type 2 diabetes mellitus: DEFENCE study. Cardiovasc Diabetol.

[17] Nabrdalik-Leśniak D, Nabrdalik K, Sedlaczek K (2021). Influence of SGLT2 inhibitor treatment on urine antioxidant status in type 2 diabetic patients: a pilot study. Oxid Med Cell Longev.

[18] Lambadiari V, Thymis J, Kouretas D (2021). Effects of a 12-month treatment with glucagon-like peptide-1 receptor agonists, sodium-glucose cotransporter-2 inhibitors, and their combination on oxidant and antioxidant biomarkers in patients with type 2 diabetes. Antioxidants (Basel).

[19] Bashier A, Khalifa AA, Rashid F (2017). Efficacy and safety of SGLT2 inhibitors in reducing glycated hemoglobin and weight in Emirati patients with type 2 diabetes. J Clin Med Res.

[20] Strojek K, Yoon KH, Hruba V, Elze M, Langkilde AM, Parikh S (2011). Effect of dapagliflozin in patients with type 2 diabetes who have inadequate glycaemic control with glimepiride: a randomized, 24-week, double-blind, placebo-controlled trial. Diabetes Obes Metab.

[21] Panikar V, Joshi SR, Deogaonkar N (2018). Efficacy of SGLT2 inhibitors as the fifth drug in the management of type 2 diabetes mellitus in Asian Indians not controlled with at least 4 oral antidiabetic drugs. J Assoc Physicians India.

[22] Calapkulu M, Cander S, Gul OO, Ersoy C (2019). Lipid profile in type 2 diabetic patients with new dapagliflozin treatment; actual clinical experience data of six months retrospective lipid profile from single center. Diabetes Metab Syndr.

